# The Green Synthesis of Cu Nanoparticles and Investigation of the Antibacterial Properties and Cytotoxicity on Multidrug-Resistant *E. coli*

**DOI:** 10.2174/0115680266379834250605135159

**Published:** 2025-06-13

**Authors:** XiaoFeng Yuan, Yu Wang

**Affiliations:** 1 Department of Pharmacy, The Second Affiliated Hospital of Harbin Medical University, Harbin Medical University, Harbin, 150086, China;; 2 Department of Otolaryngology-Head and Neck Surgery, The Second Affiliated Hospital of Harbin Medical University, Harbin Medical University, Harbin, 150086, China

**Keywords:** Multi-drug resistant, *E. coli*, Nanoparticle, Therapy, Bacteria, Cu-NPs

## Abstract

**Introduction:**

Although *E. coli* is considered a normal human microbiota, it may cause life-threatening infections such as septicemia, urinary tract infections, and enteric infections. Moreover, multidrug-resistant strains are a serious challenge in the clinic due to high mortality rates and the limited number of therapeutic options. Hence, the current study aimed to benefit from *pink rose petals* as a source of green synthesis of copper nanoparticles (Cu-NPs), to investigate the antibacterial features against multidrug-resistant *E. coli,* and to measure the cytotoxicity of Cu-NPs.

**Methods:**

*Pink rose petals* were used as a reducing agent for Cu-NP synthesis, and then XRD, zeta potential, UV-Vis, FTIR, SEM, and DLS analyses were performed to characterize the synthesized NPs. Moreover, the MIC and zone of inhibition values of Cu-NPs were measured and compared to common antibiotics. Additionally, the MTT assay was performed to assess the cytotoxicity.

**Results:**

The green synthesized Cu-NPs were spherical and uniform with a size of ~200 nm. The MIC of Cu-NPs was 1024 μg/ml on the MDR strain of *E. coli*, representing the antibacterial activity comparable to levofloxacin (p-value>0.05) but less than imipenem and trimethoprim (p-value<0.001). Moreover, the CC50 of synthesized Cu-NPs was 731.2 mg/ml and significantly lower than the studied antibiotics (p-value<0.001).

**Conclusion:**

The findings may suggest Cu-NPs as a promising antibacterial strategy against MDR strains of *E. coli*, however, further studies are encouraged to clarify the safety of optimized doses.

## 1 INTRODUCTION

*Escherichia coli*, commonly known as *E. coli*, is a species within the *Enterobacteriaceae* family. This bacterium is typically found in the human gastrointestinal tract and is part of the human normal microbiota. However, under certain circumstances, *E. coli* can become a harmful pathogen, leading to serious health issues such as systemic infections, urinary tract infections, and enteric infections [1]. Multidrug-resistant (MDR) forms of *E. coli* are considered a subset of *E. coli* strains that have evolved resistance to multiple classes of antibiotics. This form of resistance poses a substantial public health threat as a result of the increased complexity of managing infections and the higher mortality rates associated with these bacteria [1, 2].

Nanotechnology encompasses the art of manipulating matter at the atomic and molecular scales. This innovative field has the potential to revolutionize numerous sectors, particularly within the medical domain [3]. Nanotechnology and nanoparticles (NPs) can be leveraged to combat microorganisms and their multidrug resistance. For example, studies indicate that NPs containing silver [4, 5], copper [6, 4], gold [7], *etc*., are effective against multidrug-resistant bacteria with or without different antibiotics.

Copper (Cu)-NPs pose a variety of merits such as antioxidant [8-10], antifungal, antibacterial [10], and anticancer functions [11], suggesting the applicability of CuO-NPs in medicine. Indeed, Cu-NPs are recognized as a compelling treatment option to confront the complex issue of MDR infections [12]. The antibacterial capabilities of Cu-NPs, particularly in combating MDR bacteria, suggest these NPs as an ideal approach for the management of a variety of bacterial infections [12-14]. A plethora of evidence has represented priceless data supporting the antibacterial effectiveness of Cu-NPs, revealing the ability of these NPs to hinder the proliferation of pathogens such as *Klebsiella pneumoniae*, *Escherichia coli*, and methicillin-resistant *Staphylococcus aureus* strains [15].

Various techniques exist for the creation of NPs. Traditional methods like chemical and physical ones have been prevalent, however, they pose significant drawbacks, including high costs, excessive energy consumption, the potential use of toxic substances, and the generation of hazardous byproducts [16]. Hence, researchers have sought alternative methods to address these concerns. Consequently, advancements in biosynthesis or green synthesis techniques have emerged, leveraging plant extracts to reduce metal ions and create stable NPs [17]. Therefore, the current study aimed to utilize *rose petal* extract for the synthesis of Cu-NPS. Subsequently, the generated particles were evaluated through XRD, EDX, UV-Vis, FTIR, SEM, and DLS analytical methods. Upon the verification of NPs synthesis, the present study investigated the antibacterial efficacy of these prepared colloidal Cu-NPs against MDR *E. coli*. Moreover, the cytotoxicity of synthesized Cu-NPs was evaluated using mouse macrophage J774 cells.

## 2 MATERIALS AND METHODS

### 2.1 Reagents and Chemicals

Dried *pink rose petals* were collected from nearby gardens. Any remnants of soil, dust, or any other contaminants were meticulously eliminated by soaking them in deionized water. To produce initial precursors, copper sulfate (CuSO_4_.5H_2_O) and Dimethyl sulfoxide (DMSO) were obtained from Sigma (Germany). Before utilization, every piece of glassware was rigorously cleansed using a blend of concentrated hydrochloric acid (HCl) and deionized water. For the synthesis reactions and the creation of the rose petal extract, ultra-pure deionized water was used.

### 2.2 Petals Extraction and Synthesis of Cu-NPs

First, the dried petals were finely chopped and ground. Subsequently, these ground petals were mixed with 100 mL of ultra-pure deionized water and boiled for 15 minutes, resulting in a dense, syrupy mixture. Once cooled, this mixture was placed in a shaker for four hours. The resultant solution was then filtered using Whatman paper to further purify it. To eliminate any remaining microparticles, the solution was centrifuged at 500 ×g for 10 minutes. The supernatant portion was separated and preserved at a temperature of 4 degrees Celsius.

A concentrated copper sulfate solution was prepared by dissolving CuSO4 in ultra-pure deionized water to achieve a final concentration of 15.78 mM (2048 µg/ml). Subsequently, 4 milliliters of the aforementioned stock solution were combined with 36 milliliters of an extracted liquid, resulting in a diluted solution with a concentration of 1.578 mM (2048 µg/ml). The resulting solution was thoroughly mixed at room temperature for 24 hours. The final solution exhibited a brown-black hue.

### 2.3 Characterization of Cu-NPs

The produced NPs were evaluated through the application of several advanced techniques. These methods included the use of zeta potential analyzer, X-ray diffractometer (XRD), UV-visible spectroscopy, dynamic light scattering (DLS), scanning electron microscopy (SEM), and Fourier transform infrared spectroscopy (FTIR).

#### 2.3.1 Ultraviolet-Visible Absorption Spectrophotometry

1 mL of 10 mM Cu-NPs was individually dispersed in 12 mL Milli Q deionized water. Next, a chilled solution of 100 mM NaBH4 was mixed with 0.6 mL of this solution. Immediately the sample was transferred to a quartz cuvette and subjected to spectroscopic analysis over a broad wavelength range spanning 200 nm to 800 nm, at an interval of 15 min for 120 minutes. The Shimadzu UV-visible spectrometer (Model-UV-1800) was used to perform the measurements.

#### 2.3.2 Dynamic Light Scattering (DLS)

DLS is considered a nonintrusive, prompt, affordable, and sensitive method for analyzing NP sizes. In this technique, a laser source is directed through the NP suspension which results in light scattering, leading to a reduction in intensity and a change in path direction. Malvern’s zetasizer Nano S (He-Ne laser 633nm, 4mW) DLS instrument was used in this study to perform DLS measurement. In this regard, 0.1 mg of Cu-NPs was uniformly dispersed in 1 mL Milli Q water by incubating in the sonication water bath. As a result, the mean hydrodynamic diameter of the NPs was found.

#### 2.3.3 Zeta Potential Measurements

The Horiba NP size analyzer (Model: SZ-100) was utilized to measure the zeta potential of Cu-NPs. In this regard, a dispersion containing 0.1 mg of Cu-NPs was prepared by sonicating them in 1 milliliter of Milli-Q water until a homogeneous solution was achieved. The obtained suspension was analyzed using zeta potential measurements to assess the stability of NPs.

### 2.4 Antibacterial Activity of Cu-NPs

The MDR *E. coli* strain was isolated from the wastewater of local hospitals.

The Kirby-Bauer disk diffusion method was used to carry out the antibiotic susceptibility test. According to guidelines established by the Clinical and Laboratory Standards Institute (CLSI), the potency of various antibiotics was determined.

The antibiotic selection was determined in accordance with the CLSI protocols and the common antibiotic use within the study region for addressing infections stemming from gram-negative bacteria [18]. Measuring the inhibition zone diameter was performed three times in millimeters, and the mean value was calculated. The bacterial strain exhibited remarkable resistance to ten antibiotics, which belong to four antibiotic groups: fluoroquinolones, penicillin, cephalosporins, and tetracyclines. The cultivation of strains was done in a Luria-Bertani broth at a temperature of 37 °C within a shaker incubator set at 180 RPM. When the optical density (OD=600) reached 0.8, corresponding to 10^8^ CFU/mL, the bacteria were harvested *via* centrifugation at 5000 RPM for 10 min. Following this procedure, two rinses with 0.9% isotonic saline solution were performed to remove extracellular biomolecules from the target bacteria. The antibacterial capabilities of synthesized Cu-NPs were investigated against a clinical and standard strain of *E. coli* (ATCC 25922), using a Minimum Inhibitory Concentration test. The Cu-NP concentrations used in this study ranged from 2048 to 4 μg/ml, and the bacteria were cultivated in Muller-Hinton Broth media. A bacterial suspension with a concentration of 1.5 × 10^6^ CFU/ml, equal to 0.5 MacFarland, was prepared. From this suspension, 50 μl was mixed with each Cu-NP concentration. In addition, one of the samples without Cu-NPs was included as a positive control, while another without bacteria served as a negative control in the present analysis. Along with that, the MIC values of levofloxacin (a third-generation fluoroquinolone), imipenem (a carbapenem antibiotic with a broad antimicrobial spectrum), and trimethoprim (an antifolate antibiotic that belongs to the group of diaminopyrimidines), which are standard antibiotic treatments for MDR *E. coli* [19-21], were calculated.

In addition, the efficacy of synthesized NPs and selected antibiotics was analyzed by the Well Diffusion method. In this regard, the standard wells, measuring 6-8 millimeters in diameter, were crafted in MHA plates utilizing a specialized well-boring instrument. A 16-hour grown culture of bacteria was diluted to achieve a concentration of 1.5 × 10^8^ CFU/mL and streaked onto MHA plates using sterile conditions. Next, 50 μL of Cu-NPs, levofloxacin, imipenem, and trimethoprim were added into wells equal to the concentrations of the obtained MIC. The plates were incubated at 37 °C overnight and finally, inhibition zones were determined against each concentration for each sample [22].

### 2.5 The Cytotoxicity of Synthesized CuO-NPs

The cytotoxicity of Cu-NPs was measured by the 3-(4,5-dimethylthiazol-2-yl)-2,5-diphenyl-2H-tetrazolium bromide (MTT) assay and the evaluation of cytotoxicity concentration for 50% of cells (CC50). The current study performed the MTT assay to assess the proliferation and viability of mouse macrophage *J774* cells. For this purpose, 1×10^5^ mouse macrophage J774 cells were cultured in a 96-well plate. The cells were incubated for 1 day, and then the culture medium was changed. Subsequently, 10 μL of Cu-NPs was added. Upon incubation at 37°C, 5% CO2, and 95% air for 3 days, the conditioned medium was removed, and 25 μL of MTT solution (Sigma Chemical Co., Germany) was added. After incubation in the dark for 3 hours, the medium containing MTT was discarded, and 100 μL of DMSO (Merck, Germany) was added. Ultimately, the optical absorption was obtained based on the intensity of the produced blue-colored formazan using a 96-well microplate reader at 540 nm. The enhanced absorption revealed the viability of mouse macrophage J774 cells.

The concentration of Cu-NPs and antibiotics that inhibit the growth of 50% of mouse macrophage J774 cells was regarded as CC50%. The calculation of the survival rate of the studied cells was performed utilizing the following formula (1):


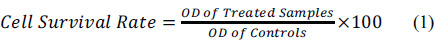


Adsorption in treated samples, which were macrophages treated with Cu-NPs, was compared with OD in controls (macrophages in medium without Cu-NPs). Moreover, the cytotoxicity of Cu-NPs was compared to the cytotoxicity of the studied antibiotics (1024, 512, 256, 128, 64, 32, 16, and 8 mg/ml). The produced color was consequently measured. All experiments were performed in triplicate.

### 2.6 Statistical Analysis

All data are expressed as mean ± standard deviation (SD). Statistical analysis was performed using SPSS 16.0. The results were analyzed by the Kruskal–Wallis test/Ordinary One-Way ANOVA test according to the normality, followed by the Tukey post hoc test at the 5% significance level (*P* < 0.05).

## 3 RESULTS AND DISCUSSION

### 3.1 Synthesis and Characterization of Cu-NPs

The current study used *pink rose petal* extract as a stabilizing agent for facile and green synthesis of Cu-NPs, which are recognized for their cost-effectiveness and eco-friendliness compared to toxic and hazardous chemicals. In fact, biomolecules extracted from plants contain phenols, peptides, and flavonoids that are documented as stabilizing and reducing agents in synthesizing metal nanoparticles [23, 24]. It appears that the natural reducing agent in *pink rose petal* extract caused the reduction of Cu, which in turn led to the aggregation and stabilization that generated Cu-NPs. Upon the addition of Cu-NP precursor to the plant extract, the color of the mixture changed to brown-black. The change of solution color is considered a representation of NPs formation [24], hence it could be assumed as an indication of the formation of Cu-NPs.

**Fig. (1):**
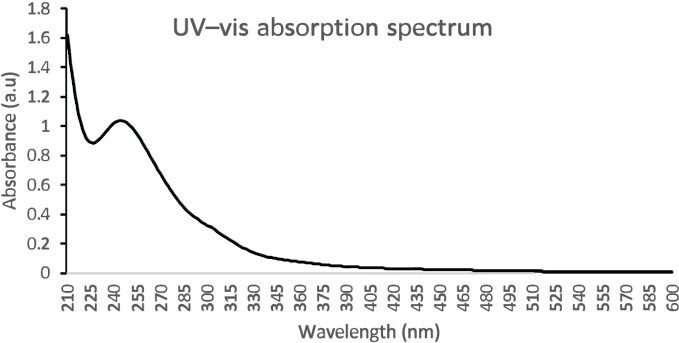
UV-visible absorption spectra of Cu-NPs at various time intervals. It is shown that Cu-NPs had an increase in peak intensity at wavelength ~ 245 nm.

UV-visible spectrometry of synthesized Cu-NPs demonstrated an increase in peak intensity at wavelength ~ 245 nm with the progress of reaction time obtained (Fig. **1**). Moreover, the time-dependent enhancement in OD of the SPR band reveals the formation of Cu-NPs. The findings of the current investigation are concordant with previous studies [25, 26] that reported a higher intensity of SPR bands in congruence with the enhanced formation of CuO-NPs. It is believed that the spectra of UV-visible absorption are considerably sensitive to the formation of NPs because these particles represent an intense peak caused by the surface plasmon excitation [27, 28].

**Fig. (2):**
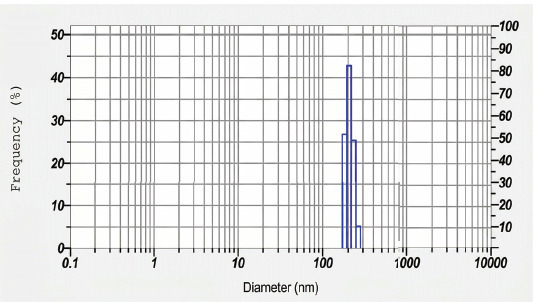
DLS analysis of Cu-NPs. The diameter of Cu-NPs was measured using the DLS technique.

**Fig. (3):**
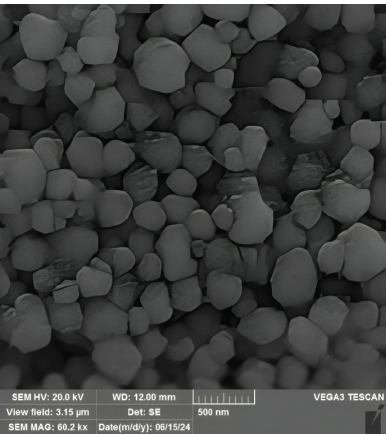
SEM image of Cu-NPs. The monoclinic structure of Cu-NPs with a mean size of 200 nm is shown.

**Fig. (4):**
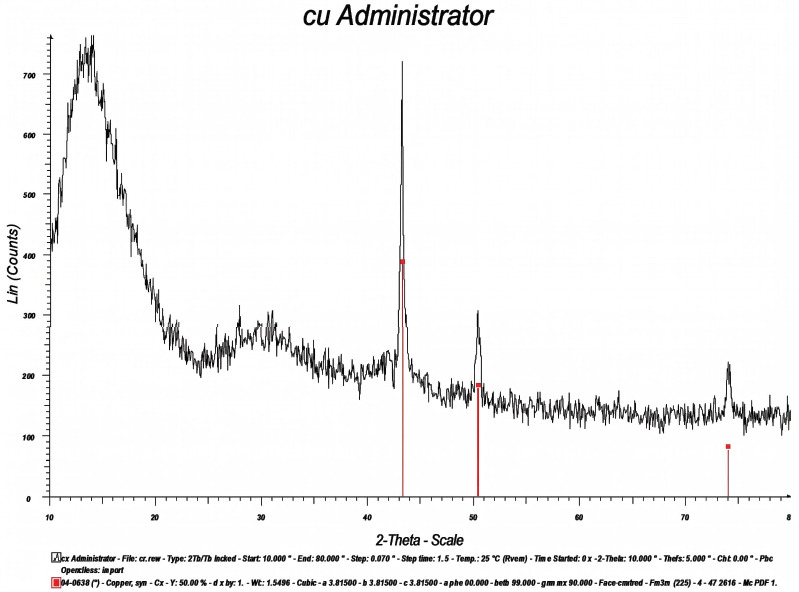
XRD patterns of green synthesized Cu-NPs. As the figure represents, Cu-NPs are in the monoclinic phase and the characteristic peaks are located at 43.2°, 50.4°, and 73.9°.

The DLS method is broadly employed for the characterization of NPs by a variety of studies [29-31]. The current study performed DLS in order to determine the hydrodynamic diameter of the green synthesized NPs. Indeed, it is documented that DLS offers valuable insights into the size distribution and mean size of synthesized NPs formed with diameters spanning a wide range, typically from nm to μm [32]. The current findings revealed that the size distribution of synthesized Cu-NPs ranged between 11 to 82 nm, and the diameter of NPs was ~ 209. Concordantly, previous studies have demonstrated that the average size of CuO-NPs was ~10-80 nm [33, 34]. Moreover, the analysis of Cu-NPs by DLS (Fig. **2**) and SEM (Fig. **3**) revealed that the synthesized NPs represent a monoclinic structure with a mean size of 200 nm. Additionally, XRD peaks confirm that the formation of Cu-NPs was in the monoclinic phase, and the characteristic peaks are located at 2 = 43.2°, 50.4°, and 73.9° (Fig. **4**).

**Fig. (5):**
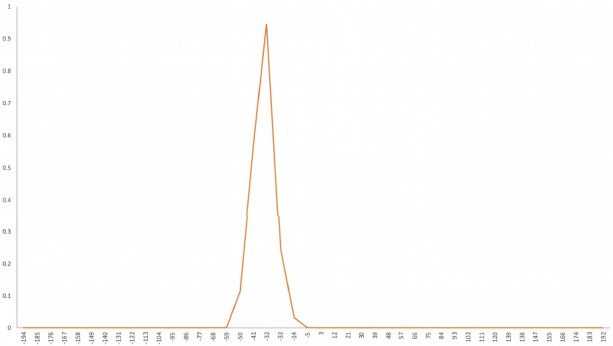
Zeta potential of Cu-NPs. Green synthesized NPs had a negative zeta potential value of -32.

It is widely documented that the potential of zeta, which primarily relies on the electrostatic repulsion between synthesized NPs, represents the net surface charge of the particles. Therefore, zeta potential is considered a major physical parameter that characterizes electrochemical equilibrium on interfaces and performs an outstanding function in the theory of NPs aggregative stability [35-37]. The findings of the present study revealed that green-synthesized Cu-NPs had a negative zeta potential value of -32 (Fig. **5**), which indicates that the surface of the NPs is negatively charged and scattered in the solution. Moreover, the negative zeta potential values prove the repulsion among the Cu-NPs and validate the strong stability and good dispersion of synthesized NPs [38, 39].

**Fig. (6):**
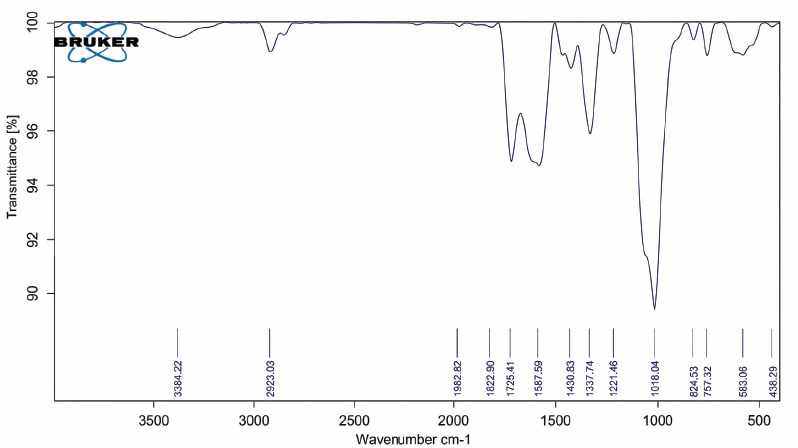
FTIR spectrum of synthesized NPs. Different peaks are as follows: peak at ~3384 cm^−1^ corresponds to O-H stretching vibrations, peak at ~2923 cm^−1^ is typically attributed to C-H stretching in alkanes, peak at ~1725 cm^−1^ corresponds to C=O stretching from carboxyl or ester groups, peak at ~1587 cm^−1^ is associated with aromatic C=C stretching, peak at ~1221 cm^−1^ represents C-O stretching, peak at ~1018 cm^−1^ can be linked to C-O or C-N stretching. Moreover, **p**eaks below 1000 cm^−1^, such as those at 824, 757, 583, and 438 cm^−1^, often correspond to metal-oxygen (Cu-O) bond vibrations.

The FTIR spectrum (Fig. **6**) provided valuable insights into the functional groups involved in the synthesis of Cu nanoparticles using pink rose plant extract. The broad peak at ~3384 cm^−1^ corresponds to O-H stretching vibrations, indicating the presence of hydroxyl groups from phenolic compounds, flavonoids, or water molecules, which play a crucial role in the reduction and stabilization of Cu nanoparticles. The peak at ~2923 cm^−1^ is typically attributed to C-H stretching in alkanes, likely from organic compounds in the rose extract. The strong peak at ~1725 cm^−1^ corresponds to C=O stretching from carboxyl or ester groups, suggesting the presence of organic acids or flavonoids. The peak at ~1587 cm^−1^ is associated with aromatic C=C stretching, which can be attributed to phenolic compounds or flavonoids in the plant extract. The peak at ~1221 cm^−1^ represents C-O stretching from alcohols, ethers, or esters, confirming the involvement of plant phytochemicals in nanoparticle formation. The peak at ~1018 cm^−1^ can be linked to C-O or C-N stretching, possibly from polysaccharides, proteins, or flavonoids. Peaks below 1000 cm^−1^, such as those at 824, 757, 583, and 438 cm^−1^, often correspond to metal-oxygen (Cu-O) bond vibrations, confirming the successful formation of Cu nanoparticles. The presence of O-H, C=O, and C=C peaks indicates that polyphenols, flavonoids, and other organic compounds from the rose extract were involved in the reduction and stabilization of Cu nanoparticles, while the Cu-O peaks in the fingerprint region validate their formation. These findings confirm that bioactive molecules from pink rose extract acted as reducing and capping agents, preventing nanoparticle aggregation and ensuring stability, thus validating the green synthesis approach.

### 3.2 Antibacterial Activity of Green Synthesized Cu-NPs

*E. coli* is considered the globally leading opportunistic pathogen that ubiquitously colonizes the gut of humans and other mammals. However, it may cause severe life-threatening infections as extra-intestinal pathogenic *E. coli* could lead to bloodstream infections that are of serious clinical concern because of a notable increase in both incidence and the fraction of isolates harboring an MDR phenotype [40-42]. Despite remarkable concerns regarding the clinical outcomes of MDR *E. coli*, a plethora of evidence has revealed that current therapeutic strategies have faced considerable challenges [43, 44]. Thereby, ongoing efforts to identify novel therapeutic options, including the synthesis and characterization of NPs, have been increasingly emphasized over the recent decade [45, 46].

After confirming the synthesis and characterization of Cu-NPs, the present study evaluated the antibacterial potential using the analysis of MIC and inhibitory zone diameter tests in normal and MDR strains of *E. coli,* and also compared the obtained results with three common antibiotics. Regarding the MIC values in standard bacteria, the results obtained are as follows: imipenem < levofloxacin, trimethoprim < Cu-NPs, whereas in the MDR strain of *E. coli*, the findings of MIC values are as follows: imipenem < trimethoprim < Cu-NPs, levofloxacin (Table **1**). MIC is the lowest concentration of a compound at which the bacteria fail to exhibit visible growth. Indeed, this metric serves as a crucial tool for precisely determining the antibiotic dosage required to inhibit bacterial proliferation effectively [24]. Although imipenem and trimethoprim antibiotics with significantly lower MIC values showed that they can inhibit the growth of bacteria in lower doses (p-value<0.001), the equal values of MIC after administration of Cu-NPs and levofloxacin may promise antimicrobial effects of synthesized NPs against MDR *E. coli*. In fact, the small size of NPs is considered an advantage that causes easy passing through the bacterial cell wall, disruption of the cell wall, and interruption of DNA structure [47, 48]. Moreover, the zone of inhibition in standard strains of bacteria is as follows: Cu-NPs < levofloxacin < trimethoprim < imipenem, while in MDR strains: levofloxacin < Cu-NPs < trimethoprim < imipenem. The present findings emphasize that the antibacterial potential of the synthesized Cu-NPs is equal to the antimicrobial efficacy of levofloxacin (p-value>0.05) but is significantly less than imipenem and trimethoprim (p-value<0.01). Nevertheless, further experiments are necessary to evaluate the promising antibacterial function against MDR *E. coli*, especially the possible cytotoxic properties of NPs in eukaryotic cells, which is discussed in the next section.

### 3.3 The Cytotoxicity of Green Synthesized Cu-NPs

MTT assay is considered a technique to validate the safety of administered doses of a specific compound and to compare its safety with similar chemicals [49]. The survival rate of mouse macrophage *J774* cells exposed to Cu-NPs compared to selected antibiotics including levofloxacin, imipenem, and trimethoprim, is shown in Fig. (**7**). The results of MTT demonstrated that the CC50% for the studied compounds is as follows: Cu-NPs (731.2 mg/ml) < trimethoprim (1622 mg/ml) < imipenem (4354 mg/ml) < levofloxacin (53564 mg/ml). The findings of the present study revealed that the rate of cytotoxicity increased as the concentration increased. Moreover, the CC50% of all studied antibiotics was notably lower than Cu-NPs (P < 0.001). Despite the appropriate efficacy of NPs according to previous extensive studies in inhibition of the growth of a variety of drug-resistant bacteria, high bioaccumulation properties and thereby the ability to damage tissues such as kidneys, liver, heart, brain, lung, and the reproductive system have faced the application of synthesized NPs with serious challenges and require careful monitoring of the level of NPs in the body [50-53]. Concordantly, the results of the current research showed that Cu-NPs are potentially able to inhibit the growth of MDR strains of *E. coli*, with an approach comparable to common antibiotics in the clinic. Nonetheless, green synthesized Cu-NPs represented more severe cytotoxicity compared to common treatment options. Thereby, further studies in this regard, particularly *in vivo* investigations, appear to be necessary to clarify both the antibacterial properties and cytotoxicity of Cu-NPs.

The current study's limitation lies in the absence of investigating the green synthesized NPs on a broader range of bacteria, particularly those exhibiting drug resistance. Additionally, it is crucial to assess the effectiveness of these NPs in animal models infected with drug-resistant *E. coli*, as well as to evaluate their potential cytotoxic effects on critical organs, including the brain, heart, liver, and kidneys. Consequently, the results of this study advocate for further research to address the aforementioned limitations.

## CONCLUSION

The results of the current investigation indicate a straightforward, rapid, and environmentally sustainable method for the green synthesis of Cu nanoparticles utilizing pink rose petals. Upon the characterization of the synthesized Cu-NPs, their efficacy in inhibiting the growth of a multidrug-resistant strain of *Escherichia coli* was assessed, showing comparable results to existing clinical treatment options, with minimum inhibitory concentration and minimum bactericidal concentration values that were favorable when compared to levofloxacin, imipenem, and trimethoprim. Nevertheless, the cytotoxicity observed in the synthesized nanoparticles within mouse macrophage J774 cells was significantly greater than that of levofloxacin, imipenem, and trimethoprim. Collectively, these results suggest that Cu nanoparticles may represent a novel and promising antibacterial therapeutic option; however, additional research is required to confirm their safety profile.

## Figures and Tables

**Fig. (7):**
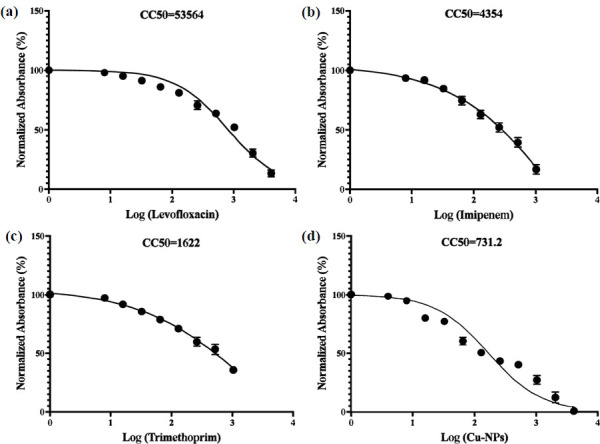
The cytotoxicity of synthesized Cu-NPs **(a)** compared to levofloxacin **(b)**, imipenem **(c**), and trimethoprim **(d)** and Log (Cu-NPs). MTT assay was performed using mouse macrophage J774 cells to reveal the cytotoxicity of the studied antibiotics and synthesized NPs.

**Table 1:** The MIC and zone of inhibition values of green synthesized Cu-NPs compared to the studied antibiotics in standard and MDR strains of E. coli.

-	**MIC**		**Zone of Inhibition (mm)**	
	**Standard *E. coli***	**MDR *E. coli***	**Standard *E. coli***	**MDR *E. coli***
***Levofloxacin***	32 mg/ml	1024 mg/ml	21.03 ± 0.41	5.77 ± 0.32
***Imipenem***	8 mg/ml	256 mg/ml	29.23 ± 1.74	12.38 ± 0.81
***Trimethoprim***	32 mg/ml	512 mg/ml	23.22 ± 1.44	8.6 ± 0.75
***Cu-NPs***	64 mg/ml	1024 mg/ml	18.33 ± 1.38	5.83 ± 0.51

## Data Availability

The data and supportive information are available within the article.

## LIST OF ABBREVIATIONS

CC50: Cell Cytotoxicity 50%
Cu: Copper
DLS: Dynamic Light Scattering
FTIR: Fourier Transform Infrared Spectroscopy
MDR: Multi Drug-Resistant
MIC: Minimum Inhibitory Concentration
NPs: Nanoparticles
SEM: Scanning Electron Microscopy
XRD: X-Ray Diffractometer
